# Conductivity enhancement of multiwalled carbon nanotube thin film via thermal compression method

**DOI:** 10.1186/1556-276X-9-451

**Published:** 2014-08-29

**Authors:** Wan-Lin Tsai, Kuang-Yu Wang, Yao-Jen Chang, Yu-Ren Li, Po-Yu Yang, Kuan-Neng Chen, Huang-Chung Cheng

**Affiliations:** 1 Department of Electronics Engineering and Institute of Electronics, National Chiao Tung University, Hsinchu 30010, Taiwan; 2 Taiwan Semiconductor Manufacturing Company, Hsinchu 30078, Taiwan

**Keywords:** Thermal compression, Carbon nanotube thin film, Carbon nanotube

## Abstract

For the first time, the thermal compression method is applied to effectively enhance the electrical conductivity of carbon nanotube thin films (CNTFs). With the assistance of heat and pressure on the CNTFs, the neighbor multiwalled carbon nanotubes (CNTs) start to link with each other, and then these separated CNTs are twined into a continuous film while the compression force, duration, and temperature are quite enough for the reaction. Under the compression temperature of 400°C and the compression force of 100 N for 50 min, the sheet resistance can be reduced from 17 to 0.9 k Ω/sq for the CNTFs with a thickness of 230 nm. Moreover, the effects of compression temperature and the duration of thermal compression on the conductivity of CNTF are also discussed in this work.

## Background

Carbon nanotubes (CNTs) have attracted much attention because of their high aspect ratio, large current capability, high mechanical strength, good chemical inertness, and high thermal conductivity [1,2]. CNT can be produced by numerous techniques such as chemical vapor deposition (CVD) method [3], arc-discharge method [4], and laser ablation method [5]. Among these methods, the CVD method is the most attractive way because of the possibility for mass production, selective growth, and well controllability in length. However, a high-temperature process is necessary for the growth of high-quality CNT via CVD method, and it is the high-temperature process that restricts some applications of CVD-grown CNTs. Therefore, the CNT solution is regarded as another way to realize a low-temperature and large-area process while the high-temperature process for the CNT growth is isolated from the deposition of CNT solution. The CNT solution can be then deposited onto a substrate to form a carbon nanotube thin film (CNTF) by various methods [6-8]. Nevertheless, the conductive resistance of a pristine CNTF is still too high to meet the requirements in practical use nowadays. And the high resistance of CNTF is majorly attributed to the defects of tubes and the junctions between CNTs as well as the latter dominated the overall conductance [9,10].

To improve the conductivity of pristine CNTF, B. Pradhan et al. [11] have introduced a composite of CNT and polymer to increase mobility for carrier transport. Y. S. Chien et al. [12] have reported the laser treatment on a Pt-decorated CNTF for enhancing the efficiency of the dye-sensitized solar cells. Also, M. Joo and M. Lee [13] applied the laser treatment on a solution-deposited CNTF for improving its conductivity. Although these reported literatures made some progress on the enhancement of conductivity for CNTFs, the complex processes, expensive equipments of laser systems, and contamination issues might restrict the applications of such reported CNTFs in future devices. In this work, a simple, low-cost, and low-temperature method of thermal compression is utilized to effectively enhance the electrical conductivity of CNTFs for the first time. The effects of compression temperature and the duration of thermal compression on the conductivity of CNTF are also discussed. In addition, a possible mechanism for the structure transformation of CNT is suggested to understand the influence of thermal compression on the conductivity of CNTF.

## Methods

The multiwalled CNTs were grown at 700°C via a thermal chemical vapor deposition system under the acetylene, nitrogen, and hydrogen ambience. The as-grown CNTs were scraped off from the substrate, and then the derived 0.03-g CNTs were suspended in a mixture of concentrated H_2_SO_4_ (95%), HNO_3_ (70%), and deionized water for 15 min at 140°C to enhance the solubility of CNTs in the following solvents. The filtered CNTs were rinsed by deionized water to remove the acidic residues. Afterwards, these acid-treated CNTs were dissolved in a mixture of ethanol and ethylene glycol and then ultrasonicated in ice bath for 3 h. After centrifugalizing, a homogeneous CNT solution with an approximate 0.5-mg/ml concentration of CNTs was sprayed onto glass substrates (Eagle 2000, Corning Display Technologies Taiwan Co., Ltd, Taipei, Taiwan) at 200°C to form the CNTFs. The thickness of CNTF could be adjusted by varying the spray times, and therefore, the 110-nm-thick and 230-nm-thick CNTFs on the glass substrates were obtained, respectively. Subsequently, two glass substrates, one was deposited with CNTF and the other was a bare glass substrate, were face-to-face compressed with a force of 100 N. The thermal compression temperature was varied from room temperature to 400°C, and the compression duration changed from 0 to 50 min.

## Results and discussion

**Figure 1:**
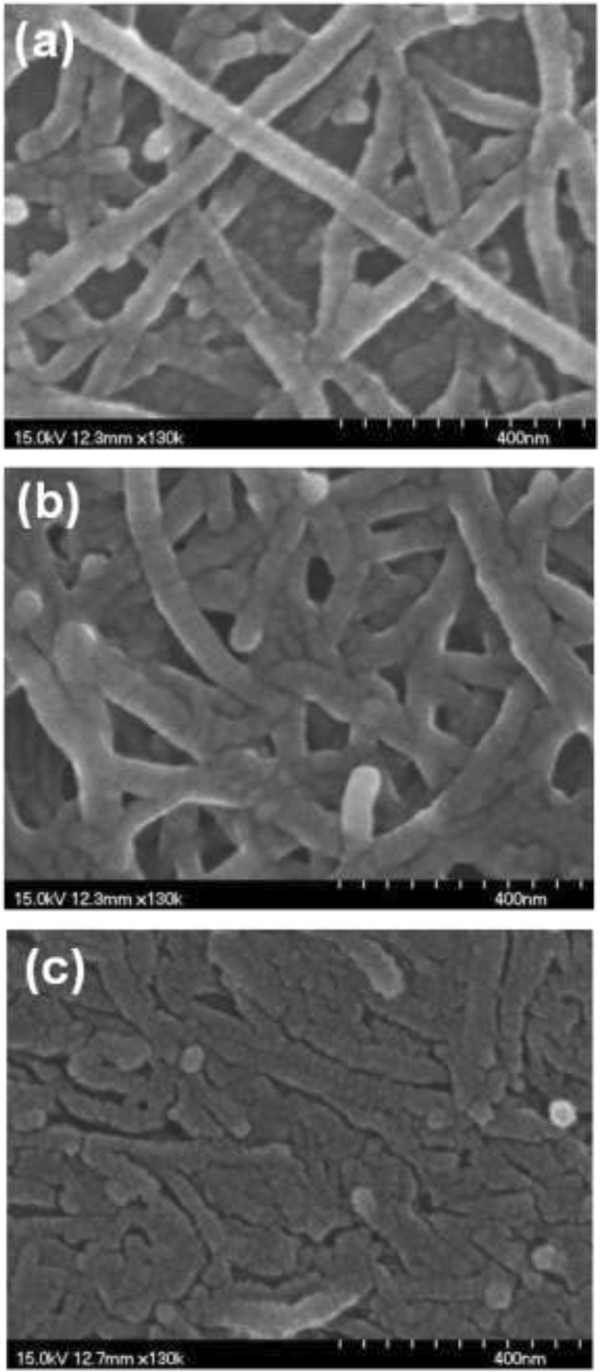
**SEM images of the morphological variations for the as-sprayed and thermally compressed CNTFs.** SEM images of **(a)** as-sprayed CNTF **(b)** under the compression force of 100 N at 200°C for 50 min and **(c)** under the compression force of 100 N at 400°C for 50 min.

**Figure 2:**
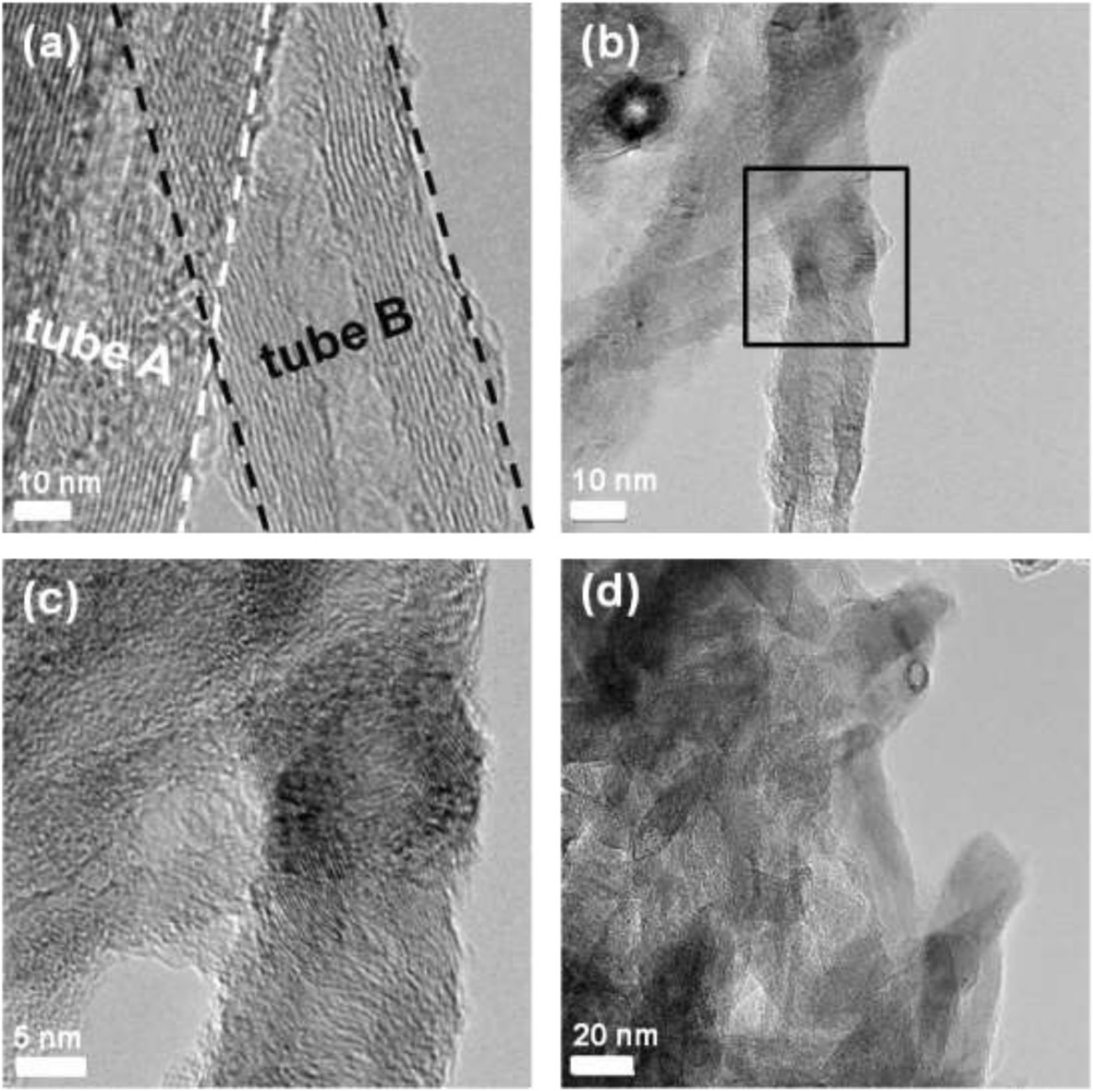
**TEM images of the as-sprayed and thermally compressed CNTs.** The high-resolution images of **(a)** the as-sprayed CNTs and **(b)** the CNTs after the thermal compression with the compression force of 100 N at 200°C for 50 min. **(c)** The magnified image of the squared region in **(b)** and **(d)** the CNTF after the thermal compression with the compression force of 100 N at 400°C for 50 min.

The field emission scanning electron microscopy (FE-SEM) images of the morphological variations for the as-sprayed CNTF and thermally compressed ones are shown in Figure 1. The CNTs in the as-sprayed CNTF can be recognized individually and distributed arbitrarily with the wire shape, as exhibited in Figure 1a. After the thermal compression with the compression force of 100 N at 200°C for 50 min, the neighbor CNTs seem to be intertwined with each other and each CNT is hard to be distinguished, as shown in Figure 1b. Once the compression temperature reaches to 400°C, the wire-shaped CNTs no longer exist and the CNTs merge into a continuous film, as shown in Figure 1c. Moreover, from the color contrast in Figure 1c, the surface of CNTF compressed at 400°C becomes much smoother than others.To further realize the effect of the thermal compression on the structure of CNT, the high-resolution transmission electron microscopy (TEM) is executed to analyze the as-sprayed CNTs and thermally compressed ones. For the as-sprayed CNTs shown in Figure 2a, two stacked CNTs are exhibited with the regular and coaxial multiwalled structures, as indicated by the dashed lines. Furthermore, it is easy to distinguish each wall structure even though one CNT stacks on the other. After the thermal compression with the compression force of 100 N at 200°C for 50 min, as shown in Figure 2b, the multiwalled structures of CNTs at the joints are hard to be distinguished, indicating that two neighbor CNTs start to link with each other. The magnified image of the squared region in Figure 2b is also demonstrated in Figure 2c, and the multiwalled structures of CNTs at the joints twist and some amorphous structures adhering to the surface are observed. While the compression temperature increases to 400°C, the CNTs are twined into a continuous film which is consistent with the observation in SEM analysis, as exhibited in Figure 2d.

**Figure 3:**
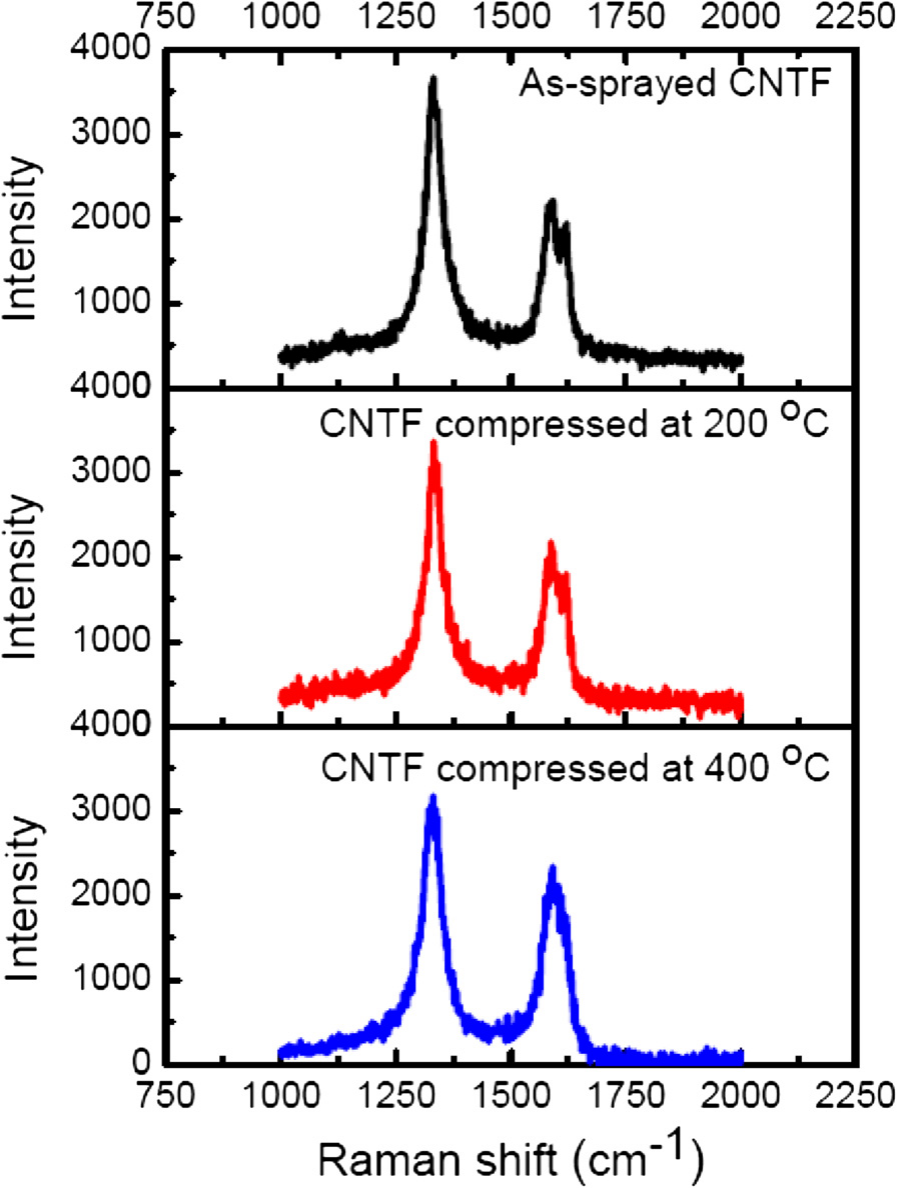
The Raman spectra of the as-sprayed CNTF and thermally compressed ones, accordingly.

**Figure 4:**
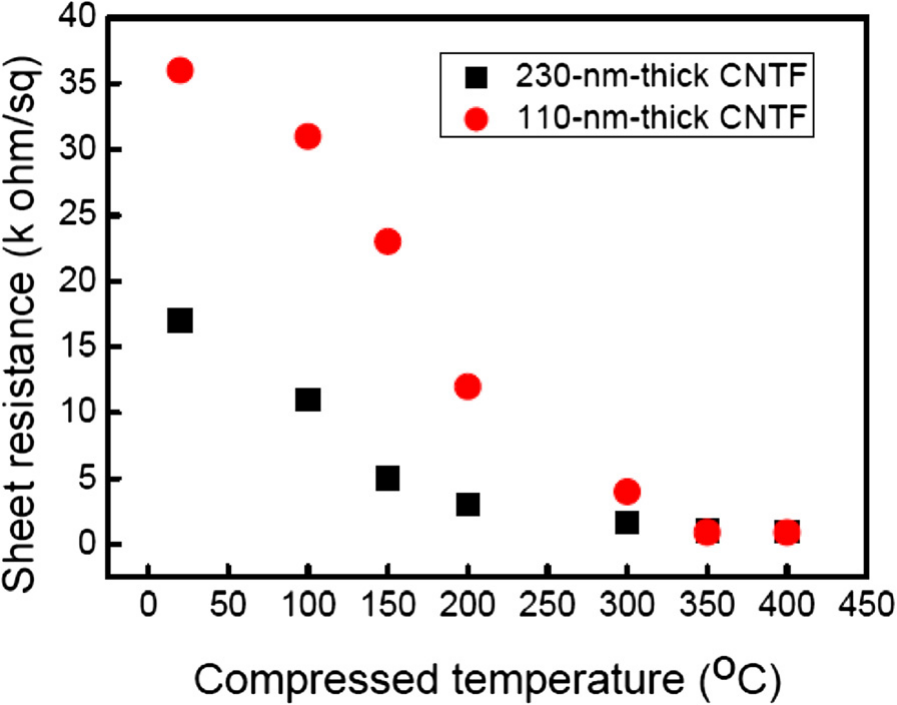
**Sheet resistance versus the compression temperature for the 110-nm-thick and 230-nm-thick CNTFs.** Sheet resistance under the compression force of 100 N for 50 min.

**Figure 5:**
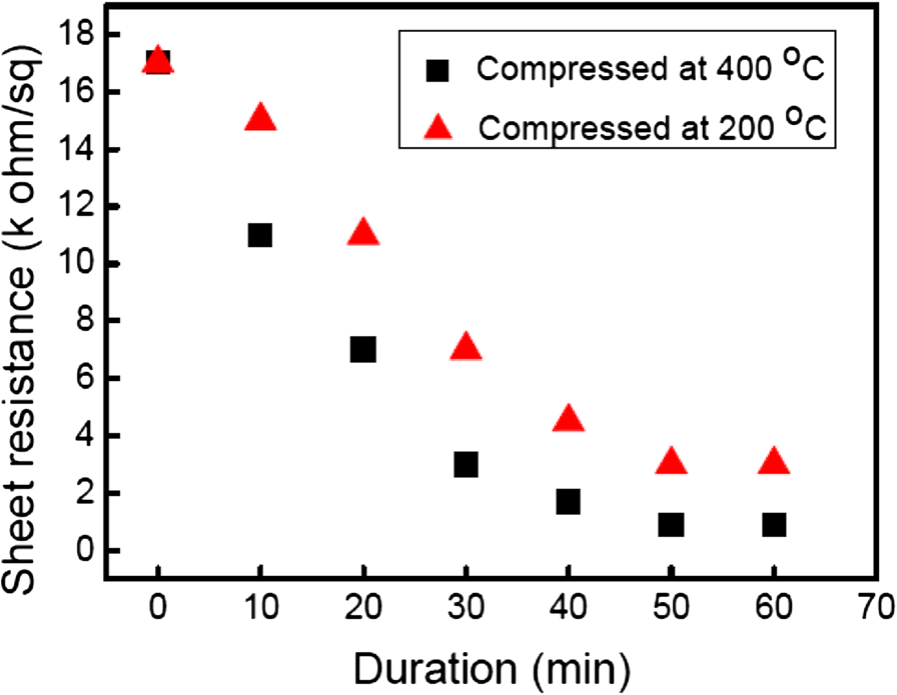
**Sheet resistance against the compression duration for the 230-nm-thick CNTFs.** Sheet resistance under the compression force of 100 N at 200°C and 400°C, accordingly.

**Figure 6:**
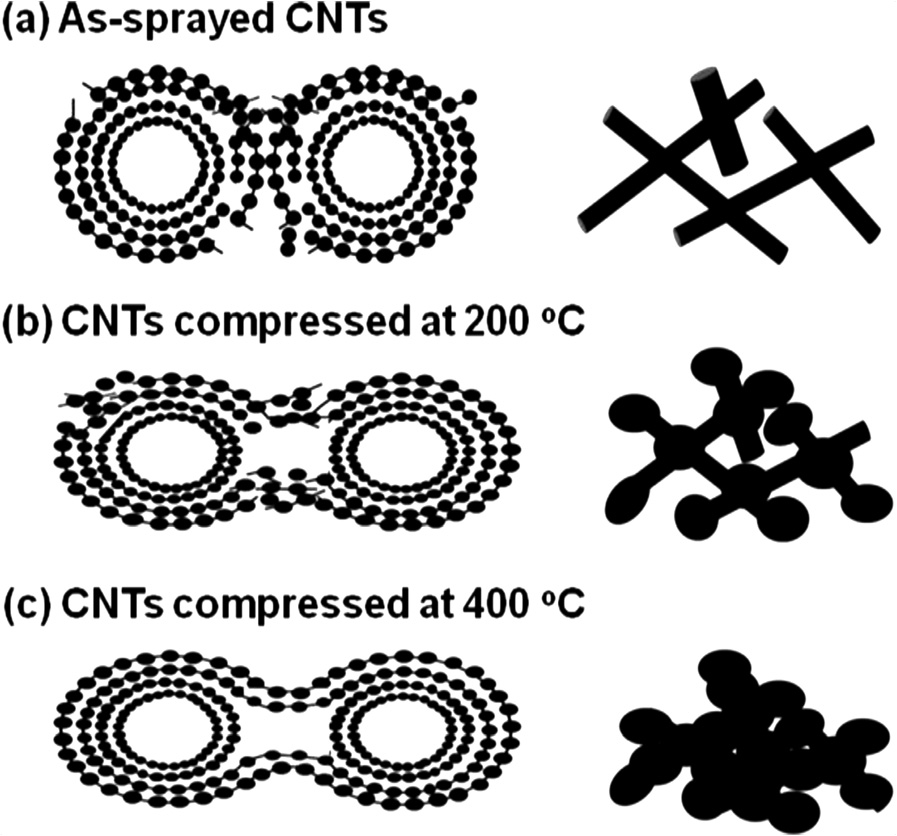
A proposed mechanism for the conductivity enhancement of CNTF after the thermal compression.

The main features of CNTFs in the Raman spectra are the disorder-induced D peak at Raman shift of 1,350 cm^-1^, and the other one is the G peak at Raman shift of 1,580 cm^-1^ corresponding to the covalent sp^2^ bonds of graphite structures, as exhibited in Figure 3. To understand the crystallinity of CNT in the CNTF after the thermal compression, the intensity ratios of D peak to G peak, *I*_D_/*I*_G_, are extracted from Figure 3. Then, the ratios of *I*_D_/*I*_G_ are about 1.79, 1.72, and 1.65 for the as-prayed CNTF and those compressed at 200°C and 400°C, accordingly. Such a high ratio of *I*_D_/*I*_G_ for the as-sprayed CNTF represents the existence of defects induced by the acid treatment. After the thermal compression at 200°C and 400°C, the ratio of *I*_D_/*I*_G_ slightly decreases, which may be attributed to the thermal annealing, and some defects on the CNTs are repaired during the compression. Furthermore, a minor band at around 1,610 cm^-1^ assigned as the D′ band is evidently observed for the as-sprayed CNTF. This band is responsible for the existence of functional groups on the CNTs after the acid treatment [14], which the CNT is treated with a mixture of concentrated H_2_SO_4_ and HNO_3_ in our case. However, the intensity of the D′ band decreases for the CNTF compressed at 200°C, and this band even disappears while the CNTF is compressed at 400°C.The sheet resistance versus the compression temperature for the 110-nm-thick and 230-nm-thick CNTFs with the compression force of 100 N for 50 min is shown in Figure 4, accordingly. It is evident that the sheet resistance decreases with the increasing of the compression temperature for these two thicknesses of CNTFs. For example, the sheet resistance decreases from 17 to 0.9 k Ω/sq as the compression temperature increases from 25°C to 400°C for the 230-nm-thick CNTFs. Moreover, the sheet resistance of the 110-nm-thick CNTF can be reduced by over 30 times after the thermal compression as the sheet resistance decreases from 36.5 to 1.1 k Ω/sq. It is also worthy to mention that the sheet resistance of the compressed CNTF seems to be the same as that of the as-sprayed CNTF at the room temperature compression, which implies that the heat plays an important role in the reduction of sheet resistance under the thermal compression.Figure 5 shows the sheet resistance against the compression duration for the 230-nm-thick CNTFs under the compression force of 100 N. The sheet resistance decreases with the increasing of the compression duration. For the compression duration of 60 min, the sheet resistance of CNTF at the compression temperature of 400°C is lower than that of the one compressed at 200°C. The initial sheet resistance for the 230-nm-thick CNTFs is 17 k Ω/sq, and the sheet resistances with the compression duration of 60 min are about 3.3 k Ω/sq for the CNTF compressed at 200°C and 0.9 k Ω/sq for the one compressed at 400°C. Although the decreasing of sheet resistance seems to be saturated after 50 min, it is suspected that the sheet resistance of CNTF can be further decreased if the compression temperature increases.A possible mechanism for the enhanced conductivity of CNTF after the thermal compression is therefore proposed. At first, there are some defects created on the surface of CNTs after the acid treatment, and the CNTs in the as-sprayed CNTF are distributed arbitrarily with the wire shape, which these CNTs contact each other at the joints without any chemical bonds, as illustrated in Figure 6a. As we know, the carriers in the length-limited CNTs need to cross a lot of junctions from one CNT to another, and then the CNTF generally attained an unsatisfied conductivity mainly attributed to the existences of these junctions at the joints of CNTs. After the thermal compression, for instance, under the compression force of 100 N at 200°C, a high pressure, close to 1 GPa at the joints of CNTs in our case, acts on CNTs, and the CNTs are squeezed and deformed, as shown in Figure 6b. With the assistance of heat, the carbon atoms around the defect sites start to bond with the neighbor carbon atoms that require a lower reaction energy. While the compression force, duration, and temperature are quite enough for the reaction, the linking of CNTs proceeds entirely, and then the CNTs are twined into a continuous film, as depicted in Figure 6c. Therefore, the carrier transports with a high conductivity after thermal compression are obtained due to the lower junction barrier at the joints of linked CNTs.

## Conclusions

In summary, the carrier transports with a high conductivity are obtained due to the lower junction barrier at the joints of linked CNTs after the thermal compression. Therefore, the sheet resistance of the 230-nm-thick CNTF decreases to 0.9 k Ω/sq with the compression temperature of 400°C and the compression force of 100 N for 50 min. Moreover, the sheet resistance of the 110-nm-thick CNTF can be reduced by over 30 times after the thermal compression to 1.1 k Ω/sq. These results for the multiwalled CNT thin films are impressive and indicate that the thermal compression method is an effective way to enhance the conductivity of CNTF. The highly conductive CNTFs after the thermal compression with the simple, low-cost, and low-temperature processes facilitate the applications of such CNTFs in the electrodes of supercapacitors, fuel cell, photovoltaic cells, and so on.

## Competing interests

The authors declare that they have no competing interests.

## Authors’ contributions

W-LT (Wan-Lin Tsai) conceived the study, participated in its experiment, and drafted the manuscript. K-YW (Kuang-Yu Wang)and Y-RL (Yu-Ren Li) participated in the experiment and material analyses. P-YY (Po-Yu Yang) performed the TEM analysis of CNTs. Y-JC (Yao-Jen Chang) participated in the experiments of thermal compression. K-NC (Kuan-Neng Chen) and H-CC (Huang-Chung Cheng) participated in its design and coordination and helped to draft the manuscript. All authors read and approved the final manuscript.

## Authors’ information

W-LT (Wan-Lin Tsai) received the B.S. degree in Electronics Engineering from National Chiao Tung University (National Chiao Tung University), Hsinchu, Taiwan, in 2004. He is currently pursuing the Ph.D. degree at the Department of Electronics Engineering in National Chiao Tung University, Hsinchu, Taiwan. His research interests include carbon nanotube and graphene in the applications of biosensor, field emission, and electronic devices.

K-YW (Kuang-Yu Wang) received the B.S. degree in Materials Science and Engineering from National Tsing Hua University (National Tsing Hua University), Hsinchu, Taiwan, in 2006. He is presently a Ph.D. student at the Department of Electronics Engineering in National Chiao Tung University (National Chiao Tung University), Hsinchu, Taiwan. His research interests include nanomaterials and biosensors.

Y-JC (Yao-Jen Chang) is currently pursuing the Ph.D. degree at the Department of Electronics Engineering in National Chiao Tung University (National Chiao Tung University), Hsinchu, Taiwan. His research interests include 3D IC, chip bonding, and electronic devices.

Y-RL (Yu-Ren Li) received the B.S. degree in Physics from National Cheng Kung University (National Cheng Kung University), Tainan, Taiwan, in 2005. She is presently a Ph.D. student at the Department of Electronics Engineering in National Chiao Tung University (National Chiao Tung University), Hsinchu, Taiwan. Her research interests include metal oxide, nanomaterials, and UV detectors.

P-YY (Po-Yu Yang) received his B.S. degree from the Institute of Display in National Chiao Tung University, Hsinchu, Taiwan, in 2007. He received the Ph.D. degree at the Department of Electronics Engineering in National Chiao Tung University (National Chiao Tung University), Hsinchu, Taiwan, in 2011. He now works in Taiwan Semiconductor Manufacturing Company, Hsinchu, Taiwan. His research interests include the applications of the devices with zinc-oxide-based nanostructures synthesized at low temperature.

K-NC (Kuan-Neng Chen) is a professor of the Department of Electronics Engineering in National Chiao Tung University (National Chiao Tung University), Hsinchu, Taiwan. He received his Ph.D. degree in Electrical Engineering and Computer Science and his M.S. degree in Materials Science and Engineering from Massachusetts Institute of Technology (MIT), respectively. Prior to the faculty position, he was a research staff member and project leader at the IBM Thomas J. Watson Research Center. His current research interests are three-dimensional integrated circuits (3D IC), through-silicon via (TSV) technology, wafer bonding technology, and heterogeneous integration.

H-CC (Huang-Chung Cheng) is a professor of the Department of Electronics Engineering in National Chiao Tung University (National Chiao Tung University), Hsinchu, Taiwan. He received the B.S. degree in physics from National Taiwan University in 1977 and the M.S. and Ph.D. degrees from the Department of Materials Science and Engineering, National Tsing Hua University (National Tsing Hua University), Hsinchu, Taiwan, in 1979 and 1985, respectively. He has published nearly 500 technical papers in international journals and conferences and also held more than 50 patents. His current research interests are in the areas of high-performance TFTs, novel nanowire devices, non-volatile memories, three-dimensional integrations, novel field emission displays, biosensors, and photoelectronic device.
